# Harmine derivative H-2-168 induces the death of *Echinococcus granulosus* by regulating mitochondrial fusion and fission

**DOI:** 10.1080/13880209.2025.2485898

**Published:** 2025-04-06

**Authors:** Yuehong Gong, Meiling Zhao, Meichi Pan, Yicong Zhao, Junpeng Liu, Hao Wen, Jianhua Wang

**Affiliations:** a Xinjiang Medical University, First Affiliated Hospital of Xinjiang Medical University, Urumqi, China; b State Key Laboratory of Pathogenesis, Prevention and Treatment of High Incidence Diseases in Central Asia, Xinjiang Medical University, First Affiliated Hospital of Xinjiang Medical University, Urumqi, China; c Department of Pharmacy, The First Affiliated Hospital of Xinjiang Medical University, Xinjiang Key Laboratory of Clinical Drug Research, Urumqi, China; d Department of Pharmacognosy, School of Pharmacy, Xinjiang Medical University, Urumqi, China; e Department of Medicine, School of Pharmacy, Xinjiang Medical University, Urumqi, China

**Keywords:** Cystic echinococcosis, *E. granulosus*, mitochondria, *Drp1*, viability

## Abstract

**Context:**

H-2-168 has pharmacological effects similar to those of harmine, with less toxicity. The health of cells and organisms depends on a delicate balance between mitochondrial fusion and fission.

**Objective:**

This study investigated the roles of H-2-168 and mitochondrial fusion and fission in *Echinococcus granulosus*.

**Materials and methods:**

Notably, *E. granulosus* were isolated from fresh sheep livers, and then treated with H-2-168 (25 μg/mL), mitochondrial division inhibitor 1 (Mdivi-1, 25 μg/mL) or the combination of H-2-168:Mdivi-1 (25 μg/mL:12.5 μg/mL). After 24 h of culture, the indices related to *E. granulosus* were measured. Additionally, Drp1 was knocked down to explore its effects on *E. granulosus* growth.

**Results:**

The EC_50_ values of H-2-168, Mdivi-1 and H-2-168:Mdivi-1 against *E. granulosus* were 44.171, 117.882 and 32.924 μg/mL, respectively. Compared with H-2-168 or Mdivi-1, the combination of H-2-168 and Mdivi-1 showed better inhibitory effects on *E. granulosus* viability, as well as increased levels of ROS and LDH, decreased ATP levels, inhibited mitochondrial activity and reduced mitochondrial membrane potential (*p* < 0.05), with the upregulation of Caspase-3, Cyt-c, Drp1, Fis1 and downregulation of Bcl-2, Mfn2 and OPA1. Additionally, *Drp1* knockdown was successfully performed in *E. granulosus*, which significantly inhibited *E. granulosus* viability (*p* < 0.05) and further downregulated Mfn2 expression induced by H-2-168.

**Discussion and conclusion:**

*Drp1* is closely associated with mitochondrial fusion and fission, and H-2-168 may promote *E. granulosus* death through disrupting the balance between mitochondrial fusion and fission.

## Introduction

Cystic echinococcosis (CE) is a chronic zoonotic parasitic disease caused by *Echinococcus granulosus*, and is a global disease (Chiodini 2023). In China, human cases of CE have been reported in at least 23 provinces (autonomous regions), and the fatality rate of this disease is 2–4%, which seriously endangers human health and economic development (Ebrahimipour et al. 2020; Ohiolei et al. 2020). Currently, the clinical treatments for CE include surgery, drug therapy, immunoprophylaxis and radiotherapy (Ferrer Inaebnit et al. 2022). Generally, surgical removal is considered the first choice of treatment, and is achieved by removing hydatid cysts and completely removing the infected parts of organs (Berto et al. 2023). However, due to the complexity of this disease, the postoperative recurrence rate is high, which not only causes serious damage to patients, but also causes substantial financial burden to them due to high treatment costs (Hammoodi et al. 2023). Goja et al. (2018) found that surgical treatment is only suitable for patients with a small number of cysts and early CE, whereas drug treatment was irreplaceable in improving the quality of life of patients with recurrent, multiple and advanced CE. Currently, the drugs recommended by the World Health Organization (WHO) for CE treatment are mainly albendazole (ABZ) and mebendazole (MBZ); however, they have the shortcomings of poor intestinal absorption and low drug concentrations in the liver (Chai et al. 2021). Additionally, both *in vitro* and *in vivo* experiments have shown that the inhibitory effects of ABZ and MBZ on parasite growth are greater than their killing effects (Das 2015), suggesting that although these drugs can prolong the life of patients and alleviate clinical symptoms, they cannot completely cure CE. Therefore, to prevent and manage CE, there is an urgent need to develop novel therapeutic drugs and targets to improve the efficacy of drug therapy.

Harmine (HM) is a tricyclic β-carboline alkaloid extracted from the seeds of *Peganum harmala* L. (Zygophyllaceae) (a local herbal medicine of Xinjiang), and has various pharmacological effects, such as antibacterial, anti-*Plasmodium*, anti-fungal, anti-oxidant, antitumor and anti-mutagenic effects (Zhang et al. 2020). Our previous studies confirmed that HM has good efficacy against CE, little hepatotoxicity and high antiparasitic activity compared to ABZ (Lu et al. 2021; Chen, Wu, et al. 2023). However, HM is severely neurotoxic, limiting its clinical use and development. Modifying the chemical structure of drugs is an effective strategy for improving their therapeutic effects and reducing their toxicity. In cooperation with Xinjiang Huashidan Pharmaceutical Co., Ltd. (Urumqi, China), our research group designed and screened the HM derivative H-2-168, which has the same parent nucleus as HM, and proved that H-2-168 is a highly effective candidate compound for CE treatment with low toxicity (Gong et al. 2020; Chen, Yan, et al. 2023). However, the mechanisms underlying the H-2-168-induced inhibition of *E. granulosus* growth and CE development remain unclear.

Previous findings from our research group showed that caspase-3 was activated when *E. granulosus* was treated with HM, and the HM derivative DH-330 significantly increased caspase-3 activity and the release of Cyt-C in *E. granulosus*, suggesting that the mitochondrial apoptosis pathway may be involved in inducing *E. granulosus* death (Gong et al. 2018; Wang et al. 2019). Mitochondria are composed of a dynamic network of continuous recombination, and the dynamic balance between mitochondrial fusion and fission is crucial for maintaining mitochondrial integrity and homeostasis and plays essential roles in regulating cell metabolism, energy production, reactive oxygen species (ROS) generation, programmed cell death and other basic cell functions (Adebayo et al. 2021). Under normal physiological conditions, mitochondrial fusion and fission are in a dynamic balance. Once this balance is disrupted, mitochondrial fusion and fission are blocked, leading to mitochondrial dysfunction and energy reduction, thereby inducing apoptosis and the occurrence of various diseases (Chan 2020; Giacomello et al. 2020). Increasing evidence has shown that mitochondrial dynamics, that is, restoring the balance between mitochondrial fusion and fission, represents a powerful therapeutic target in extensive human diseases (Whitley et al. 2019). Hu et al. demonstrated that Omentin1 helps maintain mitochondrial dynamic homeostasis and activates mitochondrial autophagy by upregulating SIRT3/FOXO3a signal transduction, thereby improving myocardial infarction-induced heart failure and myocardial injury (Hu et al. 2022). Another study illustrated that silibinin enhanced mitochondrial fission while suppressing fusion and induced the apoptosis of breast cancer cells through mitochondrial fission-induced autophagy (Si et al. 2020). However, whether H-2-168 induces *E. granulosus* apoptosis by regulating mitochondrial fusion and fission remains unclear.

In this study, *E. granulosus* was isolated from fresh sheep livers infected with *E. granulosus* and then treated with H-2-168 and mitochondrial fusion and fission inhibitor (mitochondrial division inhibitor 1, Mdivi-1) to investigate the specific roles of H-2-168 and mitochondrial fusion and fission in the growth of *E. granulosus*. Additionally, mitochondrial fission depends on Drp1, which affects the rate of mitochondrial energy synthesis (Schmitt et al. 2018; Zerihun et al. 2023). Therefore, we explored the effects and underlying mechanisms of Drp1 in mitochondrial fusion and fission in *E. granulosus*. Our results may provide novel therapeutic drugs and targets for the prevention and treatment of CE.

## Materials and methods

### Collection and culture of *E. granulosus*

The gravid proglottid of *E. granulosus* is excreted from the feces of canine feline animals, thereby polluting grasslands, feed and drinking water. After the sheep swallow the contaminated feed or drinking water, the gravid proglottid burrow into the blood vessels from the intestinal wall of the sheep, and parasitize tissues such as the liver and lungs along with the blood. When slaughtering sheep in a slaughterhouse, the butcher will find that sheep with hydatid disease have cystic structures on their liver, which are filled with liquid. In this study, fresh sheep liver infected with *E. granulosus* was identified by the experienced butcher, and obtained from the Hualing Slaughter Market (Urumqi, China). Under aseptic conditions, the inner capsule was removed from a sheep liver cyst, and the cyst fluid was extracted. The inner capsule was then cut and repeatedly rinsed with sterile normal saline. After precipitation, *E. granulosus* were collected. The collected *E. granulosus* were digested with 1% pepsin (pH 2.0, cat. no. 9001-75-6, Sigma, Kawasaki, Japan), filtered to remove impurities and *E. granulosus* with poor activity, and washed repeatedly with normal saline supplemented with 1% penicillin/streptomycin (cat. no. SV30010, HyClone, Logan, UT). Subsequently, the *E. granulosus* were transferred to a new culture bottle with culture medium and cultured in a constant temperature incubator at 37 °C and 5% CO_2_. RPMI-1640 (cat. no. SH30096.01, HyClone, Logan, UT) containing 10% fetal bovine serum (FBS, cat. no. A5670701, Gibco, Waltham, MA) and 1% penicillin/streptomycin was used as the culture medium.

### Experimental grouping and treatment

To select the optimal concentration of drugs and the optimum incubation time, different concentrations (0, 5, 10, 25, 50 and 100 μg/mL) of H-2-168 (Xinjiang Huashidan Pharmaceutical Co. Ltd., Urumqi, China), Mdivi-1 (cat. no. HY-15886, MCE, Monmouth Junction, NJ) and H-2-168:Mdivi-1 (25 μg/mL:12.5 μg/mL) were used to treat *E. granulosus* for 24, 48, 72, 96 and 120 h, and the survival rate of *E. granulosus* was determined.

In further experiments, the *E. granulosus* were treated with H-2-168 (25 μg/mL), Mdivi-1 and H-2-168:Mdivi-1 (25 μg/mL:12.5 μg/mL) for 24 h. Subsequently the survival rate of *E. granulosus*, the levels of ROS, ATP and lactic dehydrogenase (LDH), as well as mitochondrial fusion and fission were measured using the mentioned methods.

In addition, to explore the effects of *Drp1* on mitochondrial fusion and fission in *E. granulosus*, small interfering RNAs (siRNAs) with different sequences (siRNA-1203, -1523 and -2154) were transfected into *E. granulosus* by electroporation to construct *E. granulosus* with *Drp1* knockdown, and transfection efficacy was examined. The groups were as follows: control, negative control (NC), Drp1-siRNA, H-2-168, and H-2-168 + Drp1-siRNA groups.

### Induction of *E. granulosus* with Drp1 knockdown by electroporation and detection of transfection efficiency

The NC and Drp1-siRNA-1203, -1523 and -2154 labeled with FAM (green fluorescence) were designed, synthesized and provided by GenePharma (Shanghai, China). The sequences of NC and Drp1-siRNA-1203, -1523 and -2154 are shown in Table 1. Notably, *E. granulosus* with Drp1 knockdown was constructed by electroporation as previously described (Gong et al. 2021). Briefly, *E. granulosus* were cultured *in vitro* for 1 d, and approximately 2000 *E. granulosus* were added into 100 μL electroporation solution (200 mM glucose (cat. no. 50-99-7), 5 mM magnesium chloride (cat. no. 7786-30-3), 2 mM hydrophobic ethanol (cat. no. 64-17-5) and 20 mM Tris (cat. no. C140500010), pH = 7.4; China National Medicines Corporation Ltd., Shanghai, China). Subsequently, NC and Drp1-siRNA-1203, -1523 and -2154 were added to the electroporation solution at a final concentration of 5 μM. After electric shock with square wave in a 4-mm electric shock cup at 125 V for 20 ms, the electric shock cup was immediately put in a constant temperature incubator with 5% CO_2_ at 37 °C for 10 min, and then transferred to a 24-well plate with 1 mL culture medium for another 24 h of incubation.

**Table 1.** The sequences of negative control (NC), Drp1-siRNA-1203, 1523 and 2154, as well as the sequences of Drp1 and β-actin.

Name	Sequences (5′–3′)
Drp1-siRNA-1203	Sense: CUCCAGCUUAUUACCAAAUTT Antisense: AUUUGGUAAUAAGCUGGAGTT
Drp1-siRNA-1523	Sense: GAGGAUCAUUCAGCAUUGUTT Antisense: ACAAUGCUGAAUGAUCCUCTT
Drp1-siRNA-2154	Sense: GGGCAGCUGUAUAAGUCAUTT Antisense: AUGACUUAUACAGCUGCCCTT
Negative control	Sense: UUCUUCGAACGUGUCACGUTT Antisense: ACGUGACACGUUCGGAGAATT
Drp1	F: GTTCCTACAGTCCATCCTAAT R: CTCCATCAAGCCAGCATT
β-Actin	F: CTACCTCATGAAGATCCTGACC R: CACAGCTTCTCTTTGATGTCAC

After transfection for another 24 h, the viability of *E. granulosus* was measured by staining with methylene blue, and the green fluorescence intensity in *E. granulosus* of each group was observed using an inverted fluorescence microscope. Total RNA was isolated from *E. granulosus* in different groups using TRIzol reagent (cat. no. 15596026CN, Thermo Fisher Scientific, Waltham, MA) according to the manufacturer’s protocols. Subsequently, *Drp1* expression was determined by real-time quantitative PCR (RT-qPCR) to assess transfection efficiency. Briefly, total RNA was reverse-transcribed into cDNA using the PrimeScript™ RT Reagent Kit (cat. no. RR047Q, Takara, Beijing, China). The sequences of *Drp1* are shown in Table 1, and β-actin was used as a reference gene. RT-qPCR was performed using a SYBR^®^ Premix Ex-Taq™ II kit (cat. no. DRR081A, Takara, Beijing, China). The PCR reaction included the following steps: initial denaturation at 95 °C for 30 s; followed by 40 cycles of 95 °C for 5 s and 60 °C for 30 s. The mRNA expression of *Drp1* was calculated using the 2^−ΔΔCt^ method.

### Determination of survival rate of *E. granulosus*

*E. granulosus* were seeded into a 48-well plate at a density of 2000 *E. granulosus* per well and cultured overnight. The following day, the *E. granulosus* were subjected to different treatments, and after incubation, the *E. granulosus* were stained with 0.1% methylene blue, fixed, and sliced. The morphology and viability of *E. granulosus* were observed under an inverted microscope (Nikon, Shinagawa, Japan). The vigorous *E. granulosus* were not colored, and *E. granulosus* with decreased viability or death appeared blue. Based on the number of dead *E. granulosus* and total number of *E. granulosus*, the survival rates of *E. granulosus* in the different groups were calculated using the following formula: survival rate (%) = ((total number of *E. granulosus* − the number of dead *E. granulosus*)/total number of *E. granulosus*) × 100%.

### Determination of ROS, ATP and LDH levels in *E. granulosus*

The ROS levels in the *E. granulosus* treated with H-2-168 (25 μg/mL), Mdivi-1 and H-2-168:Mdivi-1 (25 μg/mL:12.5 μg/mL) were measured using a ROS detection kit (cat. no. S0034S, Beyotime Biotechnology, Shanghai, China). Briefly, the *E. granulosus* subjected to different treatments were washed with culture medium thrice, and then 10 μM 2′,7′-dichlorodihydrofluorescein diacetate (DCFH-DA) solution was added. After culturing for 20 min, the *E. granulosus* were exposed to laser red light at 635 nm for 5 min, and a confocal laser microscope (Leica, Wetzlar, Germany) was used to measure the DCF fluorescence signal. DCF (excitation = 488 nm, emission = 525 nm) produced by ROS induction was represented by a green fluorescence signal.

In addition, ATP and LDH contents in *E. granulosus* with different treatments were examined. For the determination of ATP levels, the *E. granulosus* with different treatments were cracked with 50 μL lysis buffer (RIPA lysis buffer:PMSF = 50:1), and were ground in a ball mill at a rate of 26 r/s for 3 min. The homogenate was incubated at 4 °C for 30 min to ensure complete tissue cleavage, and then centrifuged at 4 °C at 12,000 rpm for 5 min. The supernatant was harvested to determine ATP content using an ATP assay kit (cat. no. S0026, Beyotime Biotechnology, Shanghai, China) according to the manufacturer’s protocols. For LDH measurement, *E. granulosus* with different treatments were centrifuged, and the sediments were added with 150 μL LDH release reagent (PBS:LDH v:*v* = 10:1). After 1 h of incubation at 37 °C, the samples were centrifuged at 3000 rpm for 5 min, and the supernatant was collected for the measurement of LDH content using an LDH assay kit (cat. no. BC0685, Solarbio, Beijing, China) according to the manufacturer’s instructions.

### Localization of active mitochondria and mitochondrial membrane potential in *E. granulosus*

Notably, *E. granulosus* subjected to different treatments were washed three times with PBS and the medium was removed. Subsequently, 200 nM Mito-Tracker Red CMXRos (red probes, cat. no. C1035, Beyotime Biotechnology, Shanghai, China) solution or MitoTracker Green CMXRos (green probes, cat. no. 1049B, Beyotime Biotechnology, Shanghai, China) solution was added and the samples were cultured at 37 °C for 40 min. After discarding the Mito-Tracker Red CMXRos or Mito-Tracker Green CMXRos solution, the culture medium of *E. granulosus* at 37 °C was added, and a laser scanning confocal microscope was used to acquire the images and observe the mitochondrial red or green fluorescence.

The mitochondrial membrane potential of *E. granulosus* with different treatments was examined using a Mitochondrial Membrane Potential Assay Kit with JC-1 (cat. no. M8650, Solarbio, Beijing, China) following the manufacturer’s instructions. Briefly, *E. granulosus* with different treatments were washed with PBS three times, and then 1 mL of culture medium was added. Subsequently, the *E. granulosus* were stained with 1 mL JC-1 staining working solution for 20 min at 37 °C, and after discarding the supernatant, the sediments were washed with JC-1 dyeing buffer (1×) three times. After sealing, images of *E. granulosus* were captured using a fluorescence microscope.

### Immunofluorescence (IF) assay

The *E. granulosus* treated with H-2-168 (25 μg/mL), Mdivi-1 and H-2-168:Mdivi-1 (25 μg/mL:12.5 μg/mL) were harvested, washed with PBS three times, and fixed with 4% paraformaldehyde for 15 min. After permeabilization by 0.5% Triton X-100 at room temperature for 20 min and blocking by 10% goat serum at 37 °C for 30 min, the *E. granulosus* were incubated with primary antibodies, including anti-caspase-3 (species: rabbit, dilution: 1:1000, RRID: AB_443014, cat. no. ab13847, Abcam, Cambridge, UK), anti-Drp1 (species: rabbit, dilution: 1:1000, RRID: AB_2927423, cat. no. ab184248, Abcam, Cambridge, UK) and anti-Mfn2 (species: rabbit, dilution: 1:5000, RRID: AB_10999860, cat. no. ab124773, Abcam, Cambridge, UK) antibodies at 4 °C overnight. The following day, the fluorescent secondary antibody against rabbit (species: mouse, dilution: 1:25,000, RRID: AB_2722564, cat. no. SA00001-2; Proteintech, Wuhan, China) was added, and then the mixture was incubated at 37 °C for 1 h in the dark. After washing with PBS, and staining with DAPI for 5 min, the images of *E. granulosus* were captured using an inverted fluorescent microscope, and the fluorescence intensity was calculated using the ImageJ software (Bethesda, MD). Briefly, three fields of view in the fluorescence sections from each group were randomly selected for image acquisition. The ImageJ software (Bethesda, MD) was used to segment the green channel of the image and convert the fluorescence image into 8-bit black and white images. The protein expression region was selected based on the threshold value, and the average gray value was calculated to represent the average fluorescence intensity (Farías et al. 2015).

### Western blotting

Total protein was isolated from *E. granulosus* subjected to different treatments using 100 μL lysis buffer (RIPA:PMSF = 100:1), and quantified using a BCA assay kit (cat. no. 23227, Thermo, Waltham, MA). Subsequently, the protein samples were separated using 12.5% SDS-PAGE for 2 h, transferred to PVDF membranes, and blocked with 5% skim milk for 1.5 h. After washing, the membranes were incubated with anti-Bax (species: rabbit, dilution: 1:10,000, RRID: AB_2061561, cat. no. 50599-2-Ig, Proteintech, Wuhan, China), anti-Cyt-c (species: rabbit, dilution: 1:5000, RRID: AB_2090467, cat. no. 10993-1-AP, Proteintech, Wuhan, China), anti-Drp1 (species: rabbit, dilution: 1:1000, RRID: AB_2927423, cat. no. ab184248, Abcam, Cambridge, UK), anti-Fis1 (species: mouse, dilution: 1:25,000, RRID: AB_2881994, cat. no. 66635-1-Ig, Proteintech, Wuhan, China), anti-Mfn2 (species: rabbit, dilution: 1:5000, RRID: AB_10999860, cat. no. ab124773, Abcam, Cambridge, UK), anti-OPA1 (species: rabbit, dilution: 1:2000, RRID: AB_2810292, cat. no. 27733-1-AP, Proteintech, Wuhan, China) and anti-β-actin (species: mouse, dilution: 1:25,000, RRID: AB_2289225, cat. no. 60008-1-Ig, Proteintech, Wuhan, China) antibodies at 4 °C overnight. After washing, the membranes were incubated with goat anti-rabbit IgG/HRP or goat anti-mouse IgG/HRP (species: mouse/goat, dilution: 1:25,000, RRID: AB_2722564/RRID: AB_2722565, cat. no. SA00001-1/SA00001-2, Proteintech, Wuhan, China) secondary antibodies at room temperature for 1 h. Finally, the protein bands were developed using a Millipore ECL system (Beyotime Biotechnology, Shanghai, China) and exposed to a chemiluminescence imager. The ImageJ software (Bethesda, MD) was used to analyze the grayscale values of the protein bands, with β-actin as the reference protein.

### Statistical analysis

Data are expressed as mean ± standard deviation, and one-way analysis of variance was performed for statistical analysis using the SPSS 25.0 software (SPSS Inc., Chicago, IL). Differences were considered statistically significant for *p* < 0.05. GraphPad Prism 6.0 (GraphPad Software Inc., La Jolla, CA) was used to visualize the data.

## Results

### Screening for optimal drug concentration and optimum incubation time

**Figure 1.**
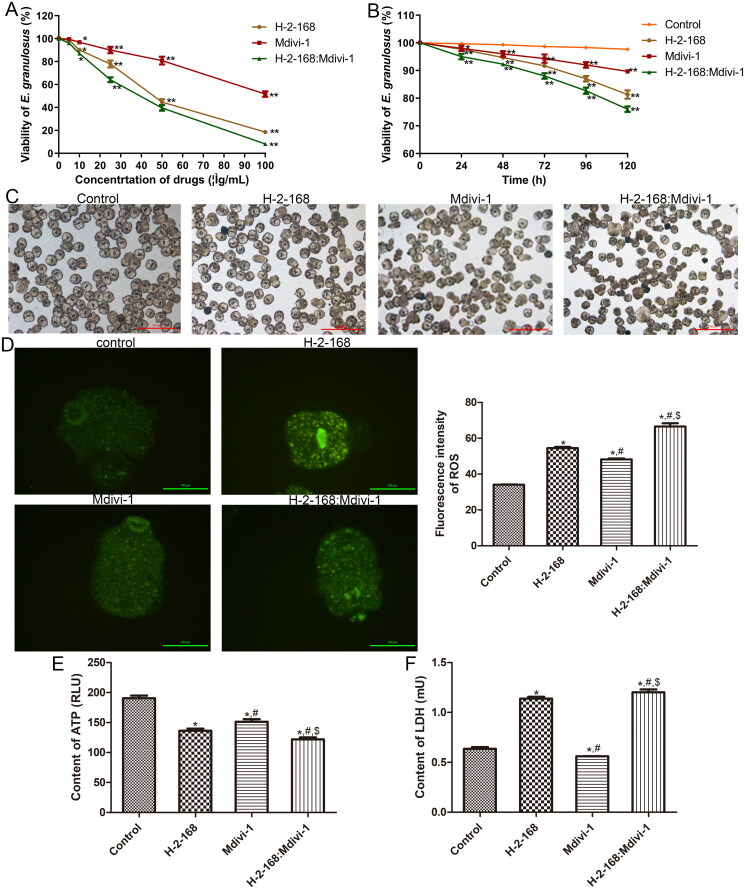
Screening for the optimal drug concentration and optimum incubation time, and the effects of H-2-168 on *Echinococcus granulosus*. (A) The viability of *E. granulosus* after treatment with different concentrations of drugs (0, 5, 10, 25, 50 and 100 μg/mL). *p < 0.05, **p < 0.01, vs. 0 μg/mL (i.e., control group). (B) The viability of *E. granulosus* subjected to different treatments after culturing for 24, 48, 72, 96 and 120 h. *p < 0.05, **p < 0.01, vs. 0 h. (C) The viability of *E. granulosus* treated with different drugs using methylene blue. Scale bar = 500 μm. (D) Reactive oxygen species (ROS) levels in *E. granulosus* treated with different drugs. (E) ATP levels in *E. granulosus* treated with different drugs. (F) Lactic dehydrogenase (LDH) levels in *E. granulosus* treated with different drugs. *p < 0.05, vs. control; ^#^*p* < 0.05, vs. H-2-168; ^$^*p* < 0.05, vs. Mdivi-1.

To screen for the optimal drug concentration and optimum incubation time, different concentrations (0, 5, 10, 25, 50 and 100 μg/mL) of H-2-168, Mdivi-1 and H-2-168:Mdivi-1 were used to treat *E. granulosus* for different times, and the viability of *E. granulosus* was measured. As shown in Figure 1(A), the results showed that the viability of *E. granulosus* gradually decreased with the increasing concentrations of the drugs, and the median effect concentration (EC_50_) values for H-2-168, Mdivi-1 and H-2-168:Mdivi-1 against *E. granulosus* were 44.171, 117.882 and 32.924 μg/mL, respectively. When the drug concentrations were 50 μg/mL and 100 μg/mL, the viability of *E. granulosus* after treatment with each drug significantly decreased (*p* < 0.05, Figure 1(A)), and a considerable number of *E. granulosus* died, which was not conducive to the follow-up experiments. No significant difference was observed in *E. granulosus* viability between the drug concentrations of 0 and 5 μg/mL (*p* > 0.05, Figure 1(A)). Therefore, 25 μg/mL of H-2-168, Mdivi-1 and H-2-168:Mdivi-1 were selected for further experiments.

In addition, after culturing for 24, 48, 72, 96 and 120 h, the viability of *E. granulosus* after different treatments was significantly inhibited compared with that of the control group (*p* < 0.05), and with increasing culture time, the viability of *E. granulosus* gradually decreased (Figure 1(B)). Therefore, the *E. granulosus* treated for 24 h were selected for subsequent analyses.

### Effects of H-2-168 on the viability of *E. granulosus* and levels of ROS, ATP and LDH

To investigate the effects of H-2-168 and Mdivi-1 on the viability of *E. granulosus*, methylene blue staining was performed. The results showed that *E. granulosus* in the control group had good activity, were not dyed blue, and had a complete and unshed head hook (Figure 1(C)). However, in the H-2-168 and Mdivi-1 groups, the outer sides of some *E. granulosus* were visibly blistered, the dead *E. granulosus* stained blue, and those alive showed reduced viability (Figure 1(C)). Additionally, in the H-2-168 + Mdivi-1 group, dead *E. granulosus* were stained blue, the worms shrank, shed their head hooks, and showed significantly reduced viability (Figure 1(C)). These results indicate that the combination of H-2-168 and Mdivi-1 may have a better inhibitory effect on *E. granulosus*.

Subsequently, the levels of ROS, ATP and LDH in *E. granulosus* subjected to different treatments were measured using the corresponding commercial kits. Compared to the control group, the fluorescence intensity of ROS in the H-2-168, Mdivi-1 and H-2-168 + Mdivi-1 groups was significantly increased (*p* < 0.05), and the fluorescence intensity of ROS was highest in the H-2-168 + Mdivi-1 group (*p* < 0.05, Figure 1(D)). The ATP contents in the control, H-2-168, Mdivi-1 and H-2-168 + Mdivi-1 groups were 190.62 ± 4.25 RUL, 136.41 ± 3.53 RUL, 151.34 ± 4.45 RUL and 121.97 ± 3.42 RUL, respectively. These results show that the ATP content in *E. granulosus* after treatment with H-2-168, Mdivi-1 and H-2-168 + Mdivi-1 was lower than that in the control *E. granulosus* (*p* < 0.05). Furthermore, compared with the H-2-168 and Mdivi-1 groups, the ATP content in the H-2-168 + Mdivi-1 group was significantly reduced (*p* < 0.05, Figure 1(E)). For LDH, its content in the control, H-2-168 and Mdivi-1 groups were 0.64 ± 0.02 mU, 1.14 ± 0.02 mU and 0.56 ± 0.0008 mU, respectively, which showed that H-2-168 significantly increased the LDH content by 78.13% compared to the control group (*p* < 0.05), whereas Mdivi-1 evidently decreased the LDH content by 12.5% compared to the control group in *E. granulosus* (*p* < 0.05, Figure 1(F)). The LDH content in the H-2-168 + Mdivi-1 group was 1.20 ± 0.03 mU, which was the highest, and was significantly increased by 87.5%, 5.26% and 114.29% compared with the control, H-2-168 and Mdivi-1 groups, respectively (*p* < 0.05, Figure 1(F)). These results suggest that the combination of H-2-168 and Mdivi-1 may have a greater effect on ROS, ATP and LDH levels in *E. granulosus*.

### Effects of H-2-168 on the activity of mitochondria and mitochondrial membrane potential in *E. granulosus*

**Figure 2.**
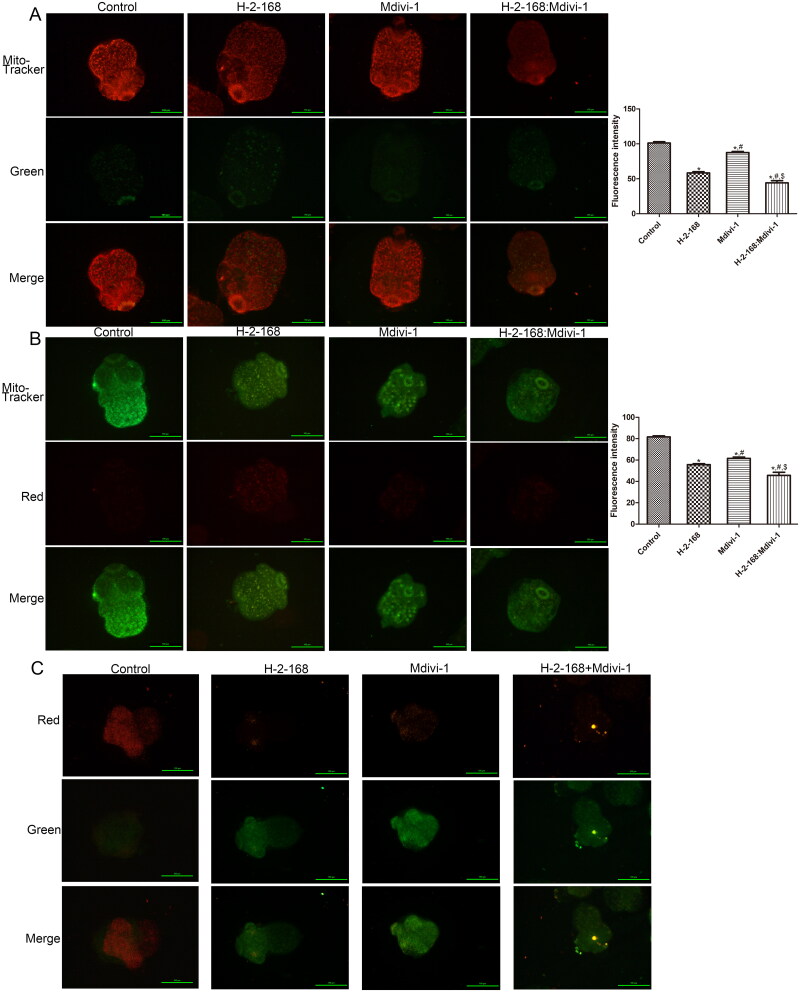
Effects of H-2-168 on mitochondrial activity and mitochondrial membrane potential in *E. granulosus*. (A) Mitochondrial activity in *E. granulosus* treated with different drugs determined using Mito-Tracker Red CMXRos (red probes). (B) Mitochondrial activity in *E. granulosus* treated with different drugs determined using Mito-Tracker Green CMXRos (green probes). (C) Mitochondrial membrane potential in *E. granulosus* treated with different drugs determined using a Mitochondrial Membrane Potential Assay Kit with JC-1. *p < 0.05, vs. control; ^#^*p* < 0.05, vs. H-2-168; ^$^*p* < 0.05, vs. Mdivi-1.

Furthermore, we determined the role of H-2-168 in mitochondrial activity and mitochondrial membrane potential in *E. granulosus*. The detection results of red (MitoTracker Red CMXRos) and green probes (MitoTracker Green CMXRos) showed that the fluorescence intensity in the H-2-168, Mdivi-1 and H-2-168 + Mdivi-1 groups was significantly lower than that in the control group (*p* < 0.05), and the fluorescence intensity in the H-2-168 + Mdivi-1 group was the lowest (Figure 2(A,B)). Subsequently, the mitochondrial membrane potential in *E. granulosus* subjected to different treatments was measured, and the normal membrane potential showed red fluorescence; when the mitochondrial membrane potential was reduced, green fluorescence was produced. The results showed that the control *E. granulosus* showed red fluorescence, which indicated normal mitochondrial membrane potential and normal-state *E. granulosus*. After treatment with H-2-168, Mdivi-1 or a combination of H-2-168 and Mdivi-1, the red fluorescence decreased, while the green fluorescence increased; the green fluorescence in the H-2-168 + Mdivi-1 group was more evident than that in the other groups (Figure 2(C)).

### Effects of H-2-168 on expression of apoptosis- and mitochondrial fusion- and fission-related proteins in *E. granulosus*

**Figure 3.**
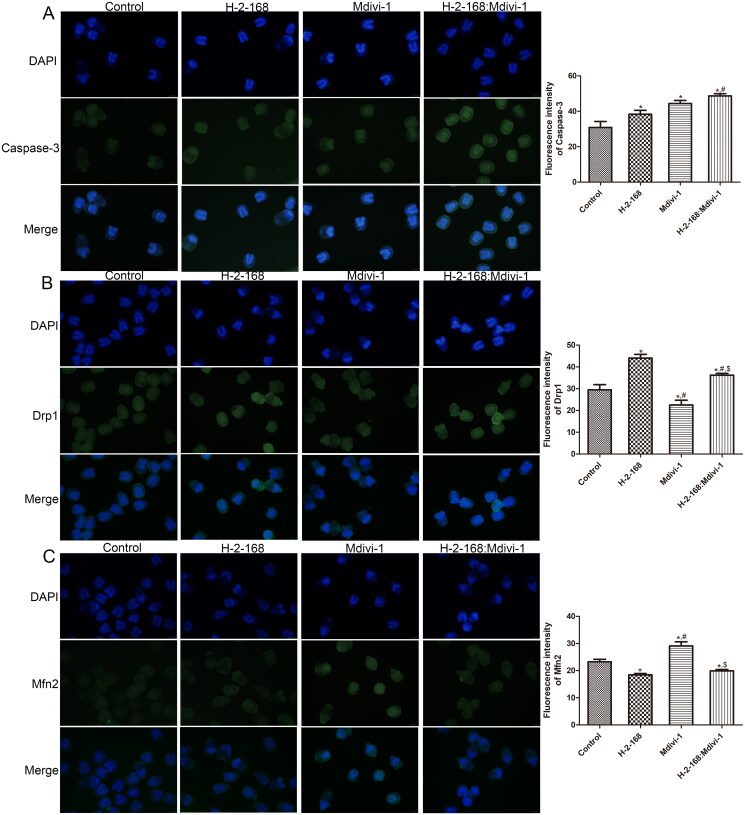
Expression of apoptosis- and mitochondrial fusion- and fission-related proteins in *E. granulosus* determined by immunofluorescence assay. (A) Fluorescence intensity of caspase-3 in *E. granulosus* treated with different drugs. (B) Fluorescence intensity of Drp1 in *E. granulosus* treated with different drugs. (C) Fluorescence intensity of Mfn2 in *E. granulosus* treated with different drugs. *p < 0.05, vs. control; ^#^*p* < 0.05, vs. H-2-168; ^$^*p* < 0.05, vs. Mdivi-1.

To explore the molecular mechanisms of H-2-168 on *E. granulosus* growth, apoptosis- (caspase-3, Bcl-2 and Cyt-c) and mitochondrial fusion- and fission-related (Drp1, Fis1, Mfn2 and OPA1) proteins in *E. granulosus* subjected to different treatments were detected by IF and western blotting. The IF results showed that the fluorescence intensity of caspase-3 in the *E. granulosus* treated with H-2-168 and Mdivi-1 and the combination of H-2-168 and Mdivi-1 was significantly higher than that in the control *E. granulosus* (*p* < 0.05), and caspase-3 fluorescence intensity was the highest in the combination group (Figure 3(A)). Compared with the control group, the fluorescence intensity of Drp1 was significantly enhanced after H-2-168 treatments (*p* < 0.05), whereas it was evidently reduced after Mdivi-1 treatment (*p* < 0.05); the combination of H-2-168 and Mdivi-1 significantly increased the fluorescence intensity of Drp1 compared with the Mdivi-1 group (*p* < 0.05, Figure 3(B)). However, the trend in Mfn2 fluorescence intensity in the different groups was opposite to that of Drp1 fluorescence intensity (Figure 3(C)).

**Figure 4.**
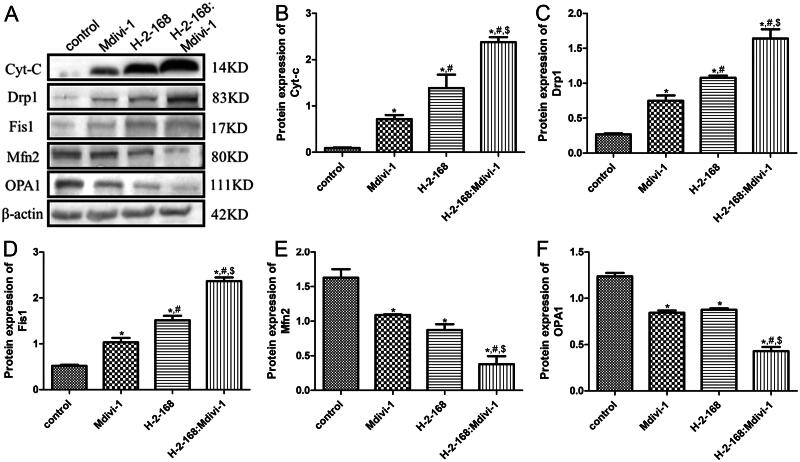
Expression of apoptosis- and mitochondrial fusion- and fission-related proteins in *E. granulosus* detected by western blotting. (A) Representative western blot bands. The protein expression of Cyt-c (B), Drp1 (C), Fis1 (D), Mfn2 (E) and OPA1 (F) in *E. granulosus* subjected to different treatments. *p < 0.05, vs. control; ^#^*p* < 0.05, vs. Mdivi-1; ^$^*p* < 0.05, vs. H-2-168.

In addition, the protein expression levels of Cyt-c, Drp1, Fis1, Mfn2 and OPA1 were examined by western blotting. Treatment of *E. granulosus* with different drugs significantly upregulated Cyt-c expression than that in the control *E. granulosus* (*p* < 0.05), and the combination of H-2-168 and Mdivi-1 exhibited better effects (Figure 4(A,B)). The expression of mitochondrial fission-related proteins (Drp1 and Fis1) was markedly upregulated in *E. granulosus* after treatments compared to that in the control *E. granulosus* (*p* < 0.05), and their expression was the highest in the H-2-168 + Mdivi-1 group (Figure 4(A,C,D)). The trend of protein expression of mitochondrial fusion-related proteins (Mfn2 and OPA1) in the different groups was opposite to that of mitochondrial fission-related proteins (Figure 4(A,E,F)).

### Transfection efficiency and selection of transfection sequences

**Figure 5.**
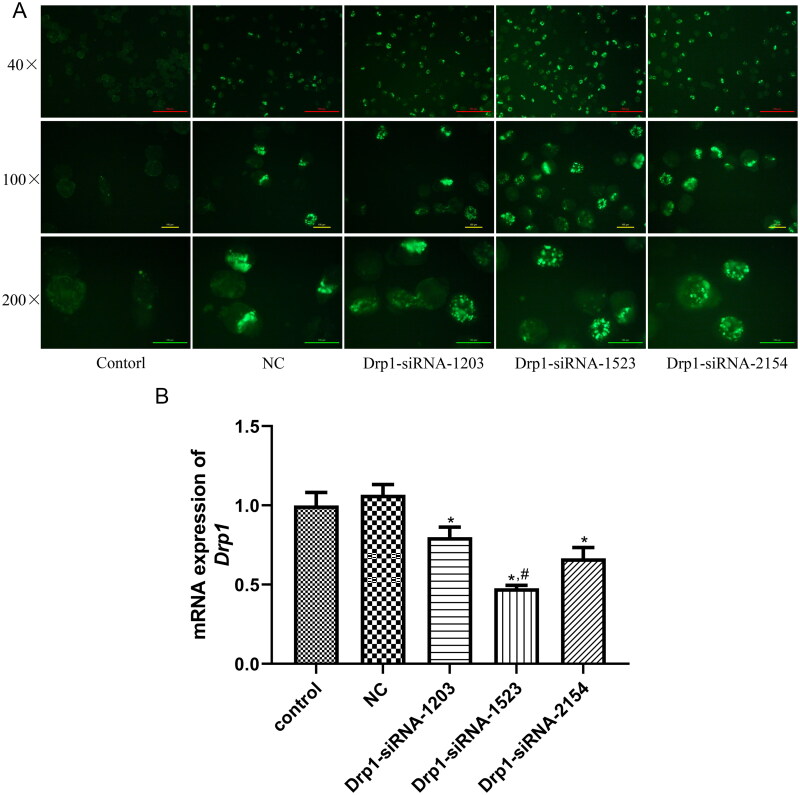
Transfection efficiency and selection of transfection sequences. (A) Green fluorescence in *E. granulosus* after electroporation with different small interference RNAs (siRNAs) observed under an inverted fluorescence microscope. (B) Expression levels of Drp1 in *E. granulosus* after transfection examined by RT-qPCR. *p < 0.05, vs. control; ^#^*p* < 0.05, vs. Drp1-siRNA-1203.

Drp1 is a mitochondrial fission-related protein closely associated with mitochondrial fusion and fission. To further investigate the roles of mitochondrial fusion and fission in *E. granulosus* growth, *E. granulosus* with *Drp1* knockdown were constructed by electroporation. After transfection, we observed no green fluorescence spots (FAM dye) in the control group; however, green fluorescent spots (FAM dye) of varying brightness were observed in the NC, Drp1-siRNA-1203, -1523 and -2154 groups (Figure 5(A)), indicating that these interference sequences with green fluorescence had been successfully introduced into *E. granulosus* by electroporation. Furthermore, green fluorescence in *E. granulosus* transfected with Drp1-siRNA-1523 was more obvious than that in the other groups (Figure 5(A)). Subsequently, the expression levels of *Drp1* after transfection were determined using RT-qPCR. The expression levels of *Drp1* in the control and NC groups were 1.00 ± 0.08 and 1.07 ± 0.06, respectively, which exhibited no significant difference (*p* > 0.05, Figure 5(B)). After transfection with Drp1-siRNA-1203, -1523 and -2154, the mRNA expression level of *Drp1* was lower than that in the control group (*p* < 0.05, Figure 5(B)). Additionally, *Drp1* mRNA expression was the lowest in the Drp1-siRNA-1523 group compared to the Drp1-siRNA-1203 and -2154 groups (*p* < 0.05; Figure 5(B)). These results implied that Drp1-siRNA-1523 had been successfully selected for transfection into *E. granulosus* in further experiments and that *E. granulosus* with Drp1 knockdown had been successfully established.

### Effects of Drp1 on *E. granulosus* viability and mitochondrial fusion- and fission-related proteins

**Figure 6.**
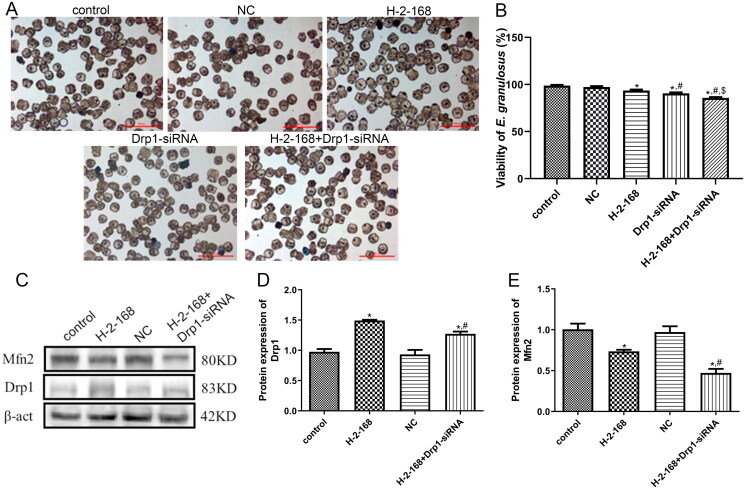
Effects of *Drp*1 on *E. granulosus* viability and on mitochondrial fusion- and fission-related proteins. (A) Representative images of *E. granulosus* subjected to different treatments stained with methylene blue. (B) Viability of *E. granulosus* subjected to different treatments. (C) Representative western blot bands. (D) Protein expression of Drp1 in *E. granulosus* subjected to different treatments. (E) Protein expression of Mfn2 in *E. granulosus* subjected to different treatments. *p < 0.05, vs. control; ^#^*p* < 0.05, vs. H-2-168; ^$^ *p* < 0.05, vs. Drp1-siRNA.

Methylene blue-stained images are shown in Figure 6(A). The viability of *E. granulosus* in the control and NC groups was 98.83 ± 0.75% and 97.33 ± 0.82%, respectively, which showed no significant difference between the control and NC groups (*p* > 0.05, Figure 6(B)). The viability of *E. granulosus* in the H-2-168, Drp1-siRNA and H-2-168 + Drp1-siRNA was 93.5 ± 1.05%, 90.5 ± 1.05% and 85.83 ± 0.75%, respectively. Relative to the control group, H-2-168 and Drp1-siRNA-1523 significantly reduced the viability of *E. granulosus* (*p* < 0.05), whereas the combination of H-2-168 and Drp1-siRNA-1523 significantly reduced the viability of *E. granulosus* (*p* < 0.05, Figure 6(B)). These results indicate that *Drp1* knockdown reduced the tolerance of *E. granulosus* to H-2-168.

In addition, we detected the protein expression of Drp1 and Mfn2 after transfection by western blotting. No significant differences were observed in Drp1 or Mfn2 protein expression between the control and NC groups (*p* > 0.05; Figure 6(C–E)). Compared to the control group, Drp1 expression was significantly upregulated after H-2-168 treatment (*p* < 0.05); however, Drp1-siRNA downregulated the H-2-168-induced Drp1 expression (*p* < 0.05, Figure 6(C,D)). The expression of Mfn2 was markedly lower after H-2-168 treatment in the group than that in the control group (*p* < 0.05); Drp1-siRNA interference further significantly downregulated its expression (*p* < 0.05, Figure 6(C,E)).

## Discussion

CE, a neglected tropical disease caused by *E. granulosus* infection, can occur through accidental ingestion of embryonic eggs from contaminated food, vegetables and water, or through direct contact with contaminated final hosts, and has considerable social and economic impacts on affected populations (Larrieu et al. 2019). HM has a wide range of pharmacological effects, including antibacterial, antiparasitic, antioxidant, antitumor and antidepressant; however, it is severely neurotoxic (Chen, Yan, et al. 2023). H-2-168, a derivative of HM, has pharmacological effects similar to those of HM, but with less toxicity. The health of cells and organisms depends on a delicate balance between mitochondrial fusion and fission (Yapa et al. 2021). In the current study, we found that the combination of H-2-168 and Mdivi-1 had better inhibitory effects on the viability of *E. granulosus*; increased ROS and LDH levels; decreased ATP levels; inhibited mitochondrial activity; reduced mitochondrial membrane potential; upregulated caspase-3, Cyt-c, Drp1, Fis1; and downregulated Bcl-2, Mfn2 and OPA1. Additionally, our study is the first to successfully construct *E. granulosus* with *Drp1* knockdown and to identify that *Drp1* knockdown further inhibited the viability of *E. granulosus* and downregulated Mfn2 in *E. granulosus*. These results indicate that H-2-168 suppressed the survival of *E. granulosus* through regulating the balance between mitochondrial fusion and fission.

Our previous study showed that H-2-168 has a remarkable effect on CE *in vitro* and *in vivo* with lower toxicity than HM (Chen, Yan, et al. 2023); however, the exact underlying mechanisms remain unclear. Mitochondria are the hubs of many important cellular processes, including ROS signaling, energy supply, cell death and survival, and heme biosynthesis (Quintana-Cabrera and Scorrano 2023). The balance between mitochondrial fusion and fission supports mitochondrial dynamics in mediating organelle morphology, cell death/viability, cell migration, metabolic plasticity and REDOX homeostasis (Picca et al. 2021). In addition to the formation of a mitochondrial network, the dynamic balance between mitochondrial fusion and fission is a checkpoint for cell death and survival (Ma et al. 2020). The energy levels change when this dynamic balance is disrupted. Kim (2023) demonstrated that HM hydrochloride inhibited the growth, invasion and migration of SK-Hep1 cells by activating PI3K/AKT signaling and inducing mitochondrial dysfunction due to oxidative stress. Another study showed that infection with *Echinococcus multilocularis* can cause changes in mitochondrial dynamics accompanied by altered energy metabolism *in vivo* and *in vitro*, with mitochondrial fission being the primary (Chaudhry et al. 2022). These reports highlight the importance of mitochondrial dynamics (especially mitochondrial fission) in *E. granulosus* infection (CE). Mdivi-1, an inhibitor of mitochondrial fission, alleviates cardiac fibrosis in the infarct boundary area after infarction by inhibiting *Drp1*-activated mitochondrial fission and oxidative stress (Ding et al. 2022). Therefore, we used Mdivi-1 and *Drp1* knockdowns to investigate whether H-2-168 could inhibit the survival of *E. granulosus* through mitochondrial fusion and fission, thereby improving CE.

ROS, by-products of cellular aerobic respiration, have been proposed as positive factors regulating multiple life activities and play essential roles in various physiological processes, such as gene expression, post-translational protein modification, cell proliferation and differentiation, homeostasis, and hypoxia adaptation (Zhang et al. 2022). Mitochondria produce ATP through oxidative phosphorylation, which provides energy for cell growth and development (Chakrabarty and Chandel 2021). A previous study showed that neuronal IFN-β is essential for mitochondrial homeostasis and metabolism, maintaining ATP levels, and preventing excessive ROS by controlling mitochondrial fission (Tresse et al. 2021). LDH can cause pyruvate to produce lactic acid through glycolysis, contributing to an imbalance in mitochondrial homeostasis and affecting cellular health (Sun et al. 2023). Mitochondrial activity and mitochondrial membrane potential are also used to reflect mitochondrial homeostasis. An imbalance in mitochondrial homeostasis can lead to energy destruction, oxidative stress and ROS overproduction. In addition, excess ROS can induce the opening of mitochondrial transition pores, resulting in impaired mitochondrial membrane potential and ultimately aggravating tissue damage (Chen et al. 2024). A study by Lu et al. (2024) observed that Mdivi-1 significantly alleviates mitochondrial dysfunction (decreased ROS levels and increased ATP levels and mitochondrial membrane potential) in ovarian granulosa cells and improves ovarian function. Another study showed that pretreatment with hirsutine ameliorated myocardial ischemia–reperfusion injury (reduced myocardial infarction area and enhanced cardiac function) by improving mitochondrial function (decreased LDH and ROS and increased ATP and mitochondrial complex activity) (Jiang et al. 2023). Based on our results, we speculate that the combination of H-2-16 and Mdivi-1 may further induce *E. granulosus* death by impairing mitochondrial function (increasing mitochondrial ROS and LDH levels and reducing ATP content and mitochondrial membrane potential).

The imbalance between mitochondrial fusion and fission can result in mitochondrial dysfunction, which manifests as a decrease in ATP production and an increase in ROS (Yu et al. 2020). We explored the expression levels of apoptosis- and mitochondrial fusion- and fission-related proteins. The combination of H-2-168 and Mdivi-1 further upregulated caspase 3, Cyt-c, Drp1 and Fis1, and downregulated Bcl-2, Mfn2 and OPA1. Caspase 3 is an apoptosis-related protein that plays a key role in regulating the growth and homeostasis of normal and malignant cells and tissues (Eskandari and Eaves 2022). Mahmoudvand et al. (2022) showed that when *Astragalus membranaceus (Fisch.) Bunge (Leguminosae)* chloroform extract was used to treat *E. granulosus* protoscolex; the caspase-3 activity and plasma membrane permeability were enhanced in a dose-dependent manner. Bcl-2, an anti-apoptotic protein, is down-regulated in *E. granulosus* infected liver tissues, thereby causing liver injury (Yang et al. 2022). Cyt c, an electron transporter in the mitochondrial respiratory chain, plays important roles in cell survival and death, and its increasing levels are widely believed to trigger cell apoptosis (Song et al. 2017). Drp1 and Fis1 are the only evolutionarily conserved mitochondrial fission proteins that interact to promote mitochondrial fission (Nolden et al. 2023). A previous study demonstrated that exposure with silver nanoparticles increased ROS levels, decreased ATP generation and mitochondrial membrane potential, and upregulated the expression of Drp1 and Fis in hippocampal HT22 cells to promote mitochondrial fragmentation via excessive mitochondrial fusion and fission, thus facilitating cell apoptosis (Chang et al. 2023). Mitochondrial fusion is overactivated in tumor organoids, and enhanced mitochondrial fusion can alter metabolism and promote tumor cell growth in liver cancer, whereas blockage of mitochondrial fusion can reduce oxygen consumption and ATP production in tumor cells (Li et al. 2020). Mfn2 and OPA1 are closely associated with mitochondrial fusion, and the knockdown of OPA1 or Mfn2 can inhibit the growth of cancer cells *in vitro* and tumor formation *in vivo*, which is related to the induction of cell apoptosis (Li et al. 2020). Taken together, the combination of H-2-168 and Mdivi-1 may promote *E. granulosus* cell death by regulating the expression of apoptosis- (caspase-3, Cyt-c and Bcl-2) and mitochondrial fusion- and fission-related proteins (Drp1, Fis1, Mfn2 and OPA1).

The maintenance of mitochondrial function and integrity is essential for the survival of normal cells. Increasing evidence has emphasized that mitochondrial homeostasis is closely associated with mitochondrial fusion and fission (Westermann 2010; Leduc-Gaudet et al. 2021). Drp1 is an indispensable core molecule that controls mitochondrial fission (Wang et al. 2020). In this study, we constructed *E. granulosus* with *Drp1* knockdown. Furthermore, we found that the tolerance of *E. granulosus* to H-2-168 was reduced after *Drp1* knockdown, and further downregulated Mfn2. Wang et al. (2020) reported that Drp1 deficiency inhibited TGF-β1-induced activization and proliferation of renal interstitial fibroblasts, whereas promoted cell apoptosis, and reduced mitochondrial fragmentation, ROS elevation and glycolytic metastasis after TGF-β1 stimulation, which may be a therapeutic target for delaying the development of chronic nephrosis. Another study showed that Drp1 was downregulated, whereas Mfn2 was upregulated in cisplatin-resistant ovarian cancer cells; Drp1 knockdown promoted cell migration and inhibited cell apoptosis, thereby contributing to the development of cisplatin resistance in ovarian cancer cells (Zou et al. 2021). Therefore, *Drp1* knockdown, similar to Mdivi-1 knockdown, may further suppress the growth of *E. granulosus* and downregulate the expression of Mfn2, thereby improving CE.

However, this study has some limitations. First, because there were substantial differences between the protoscoleces and metacestodes, further experiments should be performed to determine whether H-2-168 is effective against metacestodes. Second, toxicity studies on the combination of Mdivi-1 and H-2-168 should be conducted in the future. Additionally, further experiments using affinity chromatography coupled with mass spectrometry should be conducted to identify putative drug targets, as well as to determine whether H-2-168 can target mitochondrial fusion and fission in *E. granulosus* or in mammals.

In conclusion, the HM derivative H-2-168 may promote *E. granulosus* death by disrupting the balance between mitochondrial fusion and fission, regulating the expression of apoptosis- and mitochondrial fusion- and fission-related proteins. *Drp1* may be closely associated with mitochondrial fusion and fission. In addition, *Drp1* knockdown may further disrupt the balance between mitochondrial fusion and fission and accelerate the death of *E. granulosus*, thereby controlling CE. Our research improves our understanding of mitochondrial fusion and fission inducing the death of *E. granulosus* and lays a theoretical foundation for the prevention and treatment of CE using H-2-168 and Drp1 as a novel drug and therapeutic target, respectively.

## Data Availability

The dataset used and/or analyzed during the current study are available from the corresponding author on a reasonable request.
